# Freeze-Drying for the Reduction of Fruit and Vegetable Chain Losses: A Sustainable Solution to Produce Potential Health-Promoting Food Applications

**DOI:** 10.3390/plants14020168

**Published:** 2025-01-09

**Authors:** Dario Donno, Giovanna Neirotti, Annachiara Fioccardi, Zoarilala Rinah Razafindrakoto, Nantenaina Tombozara, Maria Gabriella Mellano, Gabriele Loris Beccaro, Giovanni Gamba

**Affiliations:** 1 Department of Agriculture, Forestry and Food Science, University of Torino, Largo Braccini 2, 10095 Grugliasco, Italy; giovanna.neirotti@unito.it (G.N.); annachiara.fioccardi@unito.it (A.F.); gabriella.mellano@unito.it (M.G.M.); gabriele.beccaro@unito.it (G.L.B.); giovanni.gamba@unito.it (G.G.); 2 Institut Malgache de Recherches Appliquées, B.P. 3833, Antananarivo 101, Madagascar; zo_ari_lala@yahoo.fr (Z.R.R.); nzara89@gmail.com (N.T.)

**Keywords:** crop losses, freeze-drying technique, dried products, health-promoting properties, phenolics, bioinformatic approach, multivariate analysis

## Abstract

Freeze-drying fresh vegetables and fruits may not only prevent post-harvest losses but also provide a concentrated source of nutrients and phytochemicals. This study focused on the phenolic composition of different freeze-dried products derived from horticultural crop remains (HCRs) in the vegetable and fruit production chain. These products may be considered as a potential health-promoting solution for preventing post-harvest fruit spoiling and losses. The total polyphenolic content (TPC) and the main phenolics were studied using high-performance liquid chromatography (HPLC) with a diode array detector (DAD). Additionally, an in vitro chemical screening of the antioxidant capacity was carried out using the Ferric Reducing Antioxidant Power (FRAP) assay. These analyses were performed together with an investigation of the correlations among phenolics and their antioxidant properties, and a bioinformatic approach was used to estimate the main potential bio-targets in human beings. Furthermore, a statistical approach using Principal Component Analysis (PCA) was carried out for a multivariate characterization of these products. Catechins, flavonols, and phenolic acids were the predominant and most discriminating classes in different products. The TPC values obtained in this study ranged from 366.86 ± 71.30 mg GAE/100 g DW (apple, MD) to 1077.13 ± 35.47 mg GAE/100 g DW (blueberry, MID) and 1102.25 ± 219.71 mg GAE/100 g DW (kaki, KD). The FRAP values ranged from 49.28 ± 2.88 mmol Fe^2+^/kg DW (apple, MD) to 80.43 ± 0.02 mmol Fe^2+^/kg DW (blueberry, MID) and 79.05 ± 0.21 mmol Fe^2+^/kg DW (kaki, KD). The proposed approach may be an effective tool for quality control and valorization of these products. This study showed that the utilization of crop remains can potentially lead to the development of new functional foods, providing additional economic benefits for farmers. Finally, the use of freeze-drying may potentially be a sustainable and beneficial solution for growers who may directly utilize this technology to produce dried products from the crop remains of their fruit productions.

## 1. Introduction

Currently, the production of food waste is a significant issue. Approximately 1.3 billion tons are lost or wasted each year from the initial steps of the supply chain to the consumer domain. However, there are variations in food waste per capita among different regions in the world [1]. For example, in North America and Europe, food waste is significantly higher (95–115 Kg/year) than in other continents. In South/Southeast Asia and Sub-Saharan Africa, however, food waste ranges from 6 to 11 Kg per year per capita, which is much lower than European and American countries, due to different factors (e.g., infrastructure for food storage, distribution to the market, economic status, socio-cultural approach to food) [2]. In recent years, several studies have focused on reducing food waste and utilizing potential crop remains and by-products. These materials are characterized by high amounts of health-promoting and nutritional compounds, and can be used to produce and develop innovative value-added food products to be transferred to the market [3]. The utilization of these by-products may not only reduce food waste but also create new and valuable products for consumption. Indeed, many residual materials and wastes, derived from food processing and rich in bioactive molecules with potential health-promoting value, can be considered a strategic tool to minimize by-products and wastes by converting them into new useful food applications [4]. In particular, fruits and vegetables are the main contributors to waste production in the food supply chain (about 44% of all food waste) because of their seasonal production and overproduction without proper utilization. It is also estimated that 25–30% of food waste specifically derives from fruit and vegetable processing, such as from pomace, peels, and seeds [1]. Moreover, fresh vegetables and fruits contain more than 80% water content, which makes them highly perishable, leading to rapid deterioration. Inadequate pre-harvest and/or post-harvest handling, as well as poor-quality processing infrastructure and insufficient marketing, contribute to significant losses, ranging from 30% (farm step) to 50% (consumer step) [5].

The remains of crop production after harvest, including by-products, wasted materials, and surplus of seasonal crops from food production and processing, contain high-quality phytochemical and nutritional substances that could be used for consumption. For this reason, it is essential to implement suitable technologies to enhance and transform these materials into viable consumable goods [1]. Post-harvest techniques are crucial for maintaining the nutritional composition, health-promoting properties, and freshness of products after harvest. However, many storage techniques require low temperatures to preserve raw materials, limiting availability in some areas and increasing costs [6]. Drying methods may offer a potential alternative to reduce losses and preserve the quality of fruits and vegetables [7,8]. In recent years, drying processes have become very important in the food industry, particularly for perishable products such as vegetables and fruits, because they can extend the shelf-life of these plant materials and inhibit the growth of microorganisms and chemical reactions, thereby preserving product quality. In the recent years, advanced drying techniques have been developed to valorize these products, allowing them to increase the sustainability and safety of the final products [2]. However, the drying processes may also cause negative effects on color, flavor and aroma, and texture, along with changes in the phytochemical and nutritional composition. These changes may disappoint the consumer requests for high-quality food items. As a result, innovative drying technologies, such as freeze-drying, have been developed to retain high levels of phytochemical, structural, and rheological properties in the dried products compared to the original fresh materials, even after removing moisture [6,9]. In particular, freeze-drying involves the extraction of water from fresh, frozen material by the sublimation process, since the frozen water is directly transformed into vapor under vacuum conditions. The obtained freeze-dried food products are highly crisp and light; moreover, this technique preserves a significant portion of aroma, original flavor, phytochemical content, and nutritional properties. Although being more costly and time-consuming if compared to traditional drying techniques, this method produces better quality products in relation to other dehydration technologies [10,11]. In any case, there is no one-size-fits-all drying method. Further studies are necessary to optimize and improve the process for different vegetables and fruits, allowing for a better diversification and availability of dried products, as well as a good economic return for producers [1].

For these reasons, drying methods are a very important processing step necessary for creating innovative edible food items. Residual edible by-products from horticultural crops and the seasonal production surplus have the potential to be used as dried food products [1,6]. Dried fruits and vegetables are a concentrated low-moisture source of nutrients and phytochemicals from fresh produce [12]. In 2018, the Middle East accounted for 28% of dried fruit consumption, followed by Europe (27%), Asia (24%), North America (13%), and other regions (8%). Global production of dried fruit increased from 2.3 million metric tons in 2009/2010 to 3.2 million metric tons in 2019/2020, with the United States, Turkey, Iran, and Saudi Arabia being the leading contributors, accounting for 16%, 15%, 12%, and 7% of the production share, respectively [13]. Both well-known dried fruits (e.g., apricots, dates, apples, prunes, figs, pears, mulberries, raisins, and peaches) and less common ones (e.g., tomatoes, cranberries, blueberries, cherries, kiwi, kaki, strawberries, and mangoes) may or may not contain added sugars or be infused with a sugar solution before the drying process. For this reason, consuming large amounts of dried products may significantly increase the intake of simple sugars, including glucose and fructose, in the diet [14]. On the other hand, dried fruits and vegetables contain different bioactive molecules and substances, including phenolic acids (e.g., benzoic acids and cinnamic acids), flavonoids (e.g., flavonols, anthocyanins, flavones, and catechins), stilbenes, proanthocyanidins, as well as phytoestrogens (including lignans and isoflavones) and carotenoids (such as lutein and zeaxanthin, α-carotene and β-carotene, and β-cryptoxanthin) [8]. In several countries, the food-based dietary guidelines (FBDG) specifically recommend consuming two to three servings (40–60 g) of dried fruits per week [13]. Thanks to their phytochemical composition, also the after-drying processes, the dried products have a positive impact on humans due to their excellent antioxidant properties. Several studies have demonstrated the beneficial influence of consuming these products on cardiovascular health and other intermediary factors related to disease risk [12]. Moreover, different in vitro and in vivo investigations showed a favorable impact of dried vegetables and fruits and relative bioactive components on tumorigenesis as well as on the regulation of glucose and lipid metabolism [15].

Despite their potential as health-promoting food items as well as sustainable alternatives to reduce crop production losses and by-products, few data are available on the phenolic composition, relative antioxidant capacity, and biological properties of these dried products [7,16]. This research focused on dried products derived from crop remains from vegetable and fruit production as a potential solution for preventing post-harvest by-products and losses. This preliminary study aimed to present the phenolic composition of freeze-dried fruits and vegetables from six species, two traditional products (apple and peach) and four unconventional ones (tomato, blueberry, kaki, and kiwifruit); total polyphenolic content (TPC), in vitro antioxidant capacity (AOC), and the fingerprint of the main health-promoting phenolics by high-performance liquid chromatography (HPLC)–diode array detector (DAD) were studied. Correlations sought among phytochemical parameters were performed together with a bioinformatic approach to estimate the main potential bio-targets in human beings. Furthermore, a multivariate statistical approach by a Principal Component Analysis (PCA) was carried out. Finally, this study may represent a preliminary basis for the large-scale exploitation of commercial dried products and their potential use as health-promoting foods.

## 2. Results and Discussion

### 2.1. Total Polyphenolic Content (TPC) and Antioxidant Capacity (AOC)

In this study, two widely used and four less popular dried food items obtained from crop remains such as undersized fruits and soft-defective materials from fruit and vegetable production were investigated. Table 1 provides a detailed overview of the dried vegetables and fruits analyzed in this research, presenting the identification (ID) code and a photographic description for each sample.

Folin–Ciocalteu reagent assay was employed to assess the total phenolic content (TPC) of the considered dried fruits and vegetables. In spite of the potential interferences, this method was utilized as a supplementary approach to confirm and validate the findings of chromatographic analysis. The TPC values obtained in this study varied from 366.86 ± 71.30 mg GAE/100 g DW (apple, MD) to 1077.13 ± 35.47 mg GAE/100 g DW (blueberry, MID) and 1102.25 ± 219.71 mg GAE/100 g DW (kaki, KD), as reported in Table 2; tomato (PD), kiwifruit (KID), and peach (PED) showed intermediate TPC levels (about 410–525 mg GAE/100 g DW), as reported in similar studies [12,13,17]. Previous research works reported the influence of drying processes on the TPC [6]. Indeed, some studies reported that blueberries and other fruits rich in antioxidants subjected to freeze-drying showed more retention of total phenolics and relative nutritional value and health-promoting benefits as compared to traditional hot air-drying, highlighting the potential of this drying technique as a preservation method for fruits and vegetables [18]. On the other hand, in the case of tomatoes, traditional drying systems may also allow them to maintain high-quality traits if temperatures between 65 °C and 75 °C are used with final levels of moisture (up to 12%) slightly lower than freeze-drying (about 15%) [6,19].

In spite of their relatively high sugar content, a negative condition if correlated to medical problems for diabetic people [12,13], recent research has indicated that dried products exhibit a glycemic index and corresponding insulin response ranging from low (55 units and below) to moderate (56 to 69 units) levels, which is lower than expected (70 units and above) [8,20]. Some studies attributed the ability to modify the glycemic response to most of the polyphenolic classes [21], changing the previous negative assumptions on dried vegetables and fruits, and highlighting their potential benefits for individuals with diabetes who need to manage their blood sugar levels.

In any case, the main beneficial health effects on humans of phenolic compounds derive from their antioxidant properties, as reported in other studies [22,23,24]. In previous studies, several mechanisms of the antioxidant capacity of phenolics were explored, including sequential proton loss electron transfer, single-electron transfer, transition metal chelation, and hydrogen atom transfer [25]. For this reason, assessing the antioxidant capacity of dried products is very important in the evaluation of their bioactivity and ability to mitigate, prevent, and reduce health issues [26]. In this research, antioxidant capacity (AOC) was also evaluated to screen for the main phenolic compounds. Some previous studies focused on traditional dried products such as dried apple and peach, but there is a lack of scientific literature on the AOC and potentially high health benefits of dried blueberry, kiwi, tomato, and kaki fruits due to their innovative nature [7]. The results of this study consistently showed high values of antioxidant capacity of the dried products in relation to the identified polyphenols and relative action mechanisms. The FRAP values varied from 49.28 ± 2.88 mmol Fe^2+^/kg DW (apple, MD) to 80.43 ± 0.02 mmol Fe^2+^/kg DW (blueberry, MID) and 79.05 ± 0.21 mmol Fe^2+^/kg DW (kaki, KD), as indicated in Table 2, consistent with findings from other research [12,16,17]. The Ferric Reducing Antioxidant Power (FRAP) assay is a good and rapid method for AOC evaluation but the health-promoting effects of antioxidation may not be only expressed by reducing power as indicated by the FRAP assay. Indeed, this test evaluates the reduction of ferric ions (Fe^3+^)-ligand to the ferrous complex (Fe^2+^) by a non-radical single-electron transfer under acidic pH conditions to maintain iron solubility in aqueous or polar solutions, not considering radical processes related to lipid oxidation in cell membranes, as reported in similar studies [8,12,27]. A single absorption endpoint may not capture the entire reaction, as different antioxidant molecules require different times to react with Fe^3+^. Moreover, the FRAP test only measures non-enzymatic antioxidant capacity, which does not accurately represent in vivo antioxidant properties in relation to the human body [28]. Polyphenolic antioxidants may be quantified only in trace amounts systemically or in vivo because of their rapid metabolic excretion, low bioavailability, and weak solubility. Furthermore, in vivo phenolic metabolites may exhibit greater antioxidant capacity compared to the relative original molecules [29,30]. For this reason, this preliminary study may have underestimated the real dried product antioxidant capacity derived from the fast-acting antioxidant phenolics, characterized by few phenolic groups, and the slow-acting ones, characterized by many -OH substituents. Consequently, the chemical targets of in vitro FRAP assay do not fully align with the real conditions and further important in vivo studies are necessary to confirm and support these preliminary results on the health-promoting benefits and biological impacts of phenolic antioxidants in dried products.

In the present research, differences in TPC and AOC values compared to other studies on dried fruits or their fresh counterparts [14,15,31] may be due to the fine equilibrium between molecule degradation and/or concentration after dehydration or drying processes [9,12]. Indeed, extended exposure to elevated temperatures and oxygen during the drying procedures, as observed in conventional drying methods (e.g., thermal drying), may lead to oxidation and reduction in bioactive substances such as phenolics and other antioxidants. Additionally, the health-promoting attributes may also be significantly impacted by the conventional hot-air drying processes due to the elevated humidity, presence of oxygen, and high temperatures [2]. Some studies reported that the antioxidant properties of dried plant material may decrease with longer drying time because of the loss and degradation of thermosensitive antioxidant molecules such as flavonoids and phenolic acids during hot-air drying (40 °C to 70 °C) [32,33]. On the other hand, differences in total phenolic content and antioxidant capacity may not be observed throughout the process, as they are primarily influenced by a significantly high variety of phenolic molecules, some of which are still unidentified. Consequently, even with a high number of antioxidant markers, no significant variation may be observed through a multi-marker approach [12].

### 2.2. Phenolic Composition, Correlation Analysis, and Biological Target Bioinformatic Prediction

HPLC analysis was performed to define the phenolic content in the hydromethanolic extracts obtained from the relative dried products. Four classes of different polyphenolic molecules were recognized and utilized as markers (multi-markers approach), as reported in previous studies [34,35]. These categories were represented by (i) benzoic acids, including gallic and ellagic acids; (ii) cinnamic acids, including caffeic, chlorogenic, p-coumaric, and ferulic acids; (iii) flavonols, including quercetin, hyperoside, isoquercitrin, rutin, and quercitrin; and (iv) catechins, including (+)-catechin and (−)-epicatechin. To identify the compounds, the retention times and UV–Vis spectra of the standards were compared with the retention times and UV–Vis spectra of the dried product extracts at each detected wavelength. The individual phenolic quantities were determined by comparing the peak area of the extract to the peak area of standard compounds using external calibration curves.

**Figure 1:**
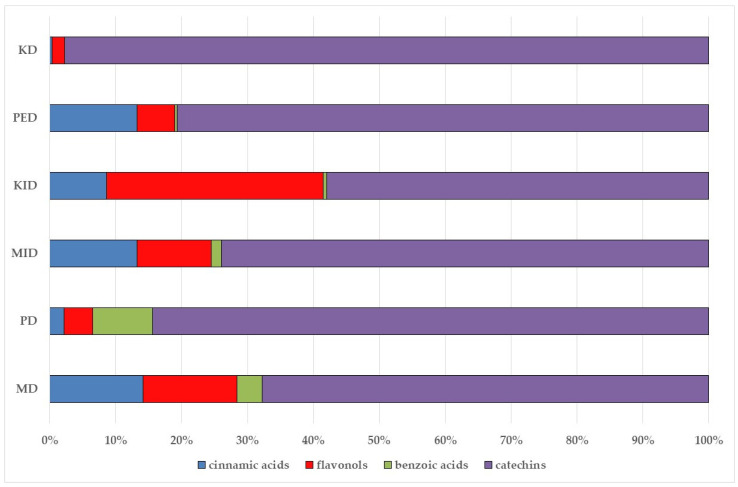
Phenolic profile of the analyzed dried products. Mean values are shown (n = 3). MD = apple; PD = tomato; MID = blueberry; KID = kiwifruit; PED = peach; KD = kaki.

The results indicated that the examined dried fruits and vegetables were a significant source of phenolics compared to similar products [9,36,37]. The contribution of each phenolic class to the total phenolic content is presented in Figure 1. Catechins were the predominant class in all the six dried products, in particular in kaki, tomato, and peach dried items (97.76%, 84.39%, and 80.67%, respectively), while phenolic acids (benzoic plus cinnamic acids) were the most significant class in blueberry (14.74%) and apple (17.93%) dried fruits. Kiwifruit and apple products exhibited a high flavonol content (about 15–30%), slightly higher than dried blueberries (about 11%).

No anthocyanins have been detected in the examined dried fruits and vegetables. It is probable that they change to phenolic acids during the drying process, as indicated in previous research [38]. Nonetheless, some studies indicated that the freeze-drying of fruits and vegetables during drying processes does not significantly alter the qualitative polyphenolic profile and that it does not impact the levels of flavonoids and other phenolics [6,39].

Each dried product showed a polyphenolic profile defined by one or more selected bioactive markers, as highlighted in Table 3.

In this study, each dried product presented a different range of phenolic compounds. Dried apples were characterized by the presence of ferulic, ellagic, and p-coumaric acids (approximately 2–15 mg/100 g DW) and quercetin (16.91 ± 0.19 mg/100 g DW), consistent with previous studies [14,16]. Cinnamic acids, notably caffeic and p-coumaric acids, were the primary phenolics in dried peaches (15.98 ± 0.25 mg/100 g DW and 20.21 ± 0.22 mg/100 g DW, respectively), along with (−)-epicatechin (152.42 ± 2.12 mg/100 g DW) and (+)-catechin (94.69 ± 3.88 mg/100 g DW). Phenolic acids as well as quercetin and quercetin-glycosides may be modified by drying processes during different technological steps, but the application of freeze-drying allows these compounds to be retained, as evidenced by other studies [6,9]. Catechins were the main polyphenols in two innovative dried products (i.e., tomato and kaki) with about 200–900 mg/100 g DW, as indicated by previous studies [12,19]. Dried tomato was also richer in gallic acid (12.11 ± 1.87 mg/100 g DW) and rutin (8.68 ± 0.27 mg/100 g DW) than the other dried products, in particular the traditional ones such as dried apple and peach. The presence of quercetin and phenolic acids, bioactive compounds with demonstrated cardioprotective properties [6], in dried fruits and vegetables is very important because they may be the key polyphenolic compounds responsible for in vitro anti-cancer activity in different parts of the human body, as recently showed by Li, et al. [41].

Four phenolic acids (caffeic, chlorogenic, ferulic, and ellagic acids), recognized as health-promoting molecules, have been identified in dried kiwifruits, particularly ferulic acid (1.99 ± 0.09 mg/100 g DW) and chlorogenic acid (1.69 ± 0.24 mg/100 g DW). Additionally, substantial levels of catechins (about 10–20 mg/100 g DW) have been also detected in these dried fruits, while flavonols (except quercetin) were found in trace amounts (<1–2 mg/100 g DW), consistent with other studies [42,43]. The HPLC results identified dried blueberry as the product with the larger variability in phenolic composition; indeed, 10 phenolics were quantified such as (−)-epicatechin (186.83 ± 2.99 mg/100 g DW), p-coumaric acid (28.27 ± 0.63 mg/100 g DW), quercetin (19.98 ± 0.36 mg/100 g DW), and quercitrin (6.83 ± 0.75 mg/100 g DW). Ferulic and ellagic acids presented smaller amounts (about 3–4 mg/100 g DW) than the main ones, while other phenolics (i.e., caffeic and chlorogenic acids, hyperoside, and rutin) were detected in trace amounts (<1–2 mg/100 g DW), the same as reported by Chen, et al. [44]. The high variability in phenolic composition may be at the base of the effects of dried blueberries on young and adult cardiovascular health [45].

**Figure 2:**
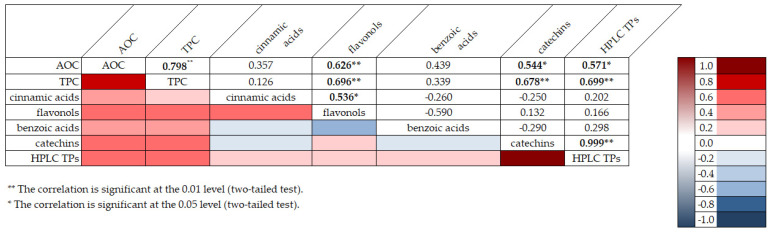
Correlation analysis among total polyphenolic content, antioxidant capacity, and phenolic classes in the considered dried products.

Pearson’s correlation coefficient (R) was used to determine the relationships among total polyphenolic content, antioxidant capacity, and phenolic classes; these coefficients were represented as values ranging from −1 to 1 (from blue to red), with zero indicating no correlation (Figure 2). The values were categorized based on the degree of correlation, as highlighted in previous studies [29,46], into strong (0.80–1.00), high (0.60–0.79), moderate (0.40–0.59), fair (0.20–0.39), and weak (0.00–0.19) correlations. Furthermore, significant correlations were presented as bold black numbers with one star for *p* < 0.05 (two-tailed test) and two stars for *p* < 0.01 (two-tailed test).

The results derived from the correlation between TPC (total phenolics by spectrophotometric assay) and AOC showed a significantly high positive correlation (R = 0.798; *p* < 0.01). Antioxidant capacity also presented a moderate positive association with HPLC TPs (total phenolics by HPLC analysis) with a value of 0.571 (*p* < 0.05). Moreover, the significant high positive correlation between TPC and HPLC TPs (R = 0.699; *p* < 0.01) confirmed that the selection of the main phenolic compounds and their quantification by HPLC fingerprinting (multi-marker approach) may be an excellent analytical strategy to assess the total phenolic content with good approximation, as already reported in previous studies [35,47].

Correlation analysis allowed us to reveal additional insights on the results of the analysis of phenolic acids and flavonoids in the dried fruits and vegetables via HPLC. Flavonols and catechins exhibited a moderate-high positive correlation with AOC by the FRAP assay (R = 0.626 at *p* < 0.01 for flavonols and R = 0.544 at *p* < 0.05 for catechins, respectively). The results indicated that these phenolic classes significantly contributed to the chemical mechanism of antioxidant capacity in the considered extracts. These two phenolic classes also showed a significant high positive correlation with TPC (R = 0.696 at *p* < 0.01 for flavonols and R = 0.678 at *p* < 0.01 for catechins, respectively), confirming them to be the main polyphenols in the dried products, as shown in the relative phenolic profile (Figure 1). Moreover, cinnamic acids and flavonols showed a significant moderate positive correlation (R = 0.536; *p* < 0.05) with each other, confirming their participation in the same biological properties (cardioprotecting and in vitro anti-cancer activity) [6,41]. On the other hand, benzoic acids showed a non-significant fair-moderate negative impact on the other phenolic classes.

The present study also carried out a preliminary bioinformatic approach by a web platform (i.e., the Swiss Target Prediction Network, http://www.swisstargetprediction.ch/, accessed on 20 September 2024) on the selected dried products to identify the potential human target proteins of the main phenolics responsible for their observed biological effects, as reported by similar studies [26,48]. The chemical structure of the main phenolics and the preliminary identification of their molecular targets by using the Swiss Target Prediction platform were reported in Table 4 and Table 5.

By evaluating the chemical structure of these compounds and the relative target proteins, this study aimed to provide a preliminary understanding of the health-promoting properties of the considered dried products. Further in vitro and in vivo analyses on the metabolism and bioavailability of these phenolic molecules in humans may be performed to confirm these results. Indeed, the ADME (absorption, distribution, metabolism, and excretion) of polyphenols in the human body is not well understood due to limited data [49]. Information from in vitro models should be correlated with human studies and animal models to fully understand the effects of these compounds. It may be crucial to identify the metabolites of these molecules after urinary excretion in order to demonstrate their potential impact on human health. Cellular models could also play a significant role in understanding the biological relevance and potential health benefits of these molecules.

For example, the platform presented that p-coumaric acid (quantified in dried apple, blueberry, and peach) targeted a large group of lyase enzymes (e.g., carbonic anhydrase group) and several oxidoreductases (e.g., aldose reductase and arachidonate 5-lipoxygenase), being involved in many cellular processes related to anti-inflammatory and antiallergic responses [50,51,52]. Moreover, it targets the estrogen receptor beta participating in the growth, differentiation, and function of the reproductive system [53]. Similar to p-coumaric acid, ferulic acid (quantified in dried tomato, kiwifruit, and kaki) targeted a carbonic anhydrase (i.e., CA2), an enzyme involved in carbon dioxide transport and acid–base balance with an important action in pH regulation, suggesting its potential anti-inflammatory properties [54,55]. Moreover, the web platform showed that ellagic acid (quantified in dried apple and kiwifruit) and quercetin (quantified in dried apple, peach, kiwifruit, and kaki) interacted with the G protein-coupled receptor 35 (GPR35) in the immune cells and digestive tract [48]. This interaction may contribute to the neuroprotective and anti-inflammatory properties of these compounds, as well as potentially impacting digestive functions [56]. The platform output also highlighted the interaction of quercetin with cytochrome P450, as reported in the literature [57,58]. These preliminary findings highlighted the potential health-positive benefits of these phenolics, particularly their anti-inflammatory effects.

The target proteins of (+)-catechin and (−)-epicatechin in the human body were also evaluated by the same web platform (Table 5). The main biological targets of these compounds (e.g., a large class of enzymes, membrane receptors, cytosolic and nuclear proteins, ion channels, and transcription factors) confirmed their positive influence on human health, such as their ability against allergies, inflammation, microbes and viruses, and even cancer [59]. Moreover, catechins may increase the effectiveness and absorption of other health-promoting substances, such as some molecules in biocosmetics and/or functional foods, into human skin and bodies [60].

### 2.3. Multivariate Approach and Principal Component Analysis

**Figure 3:**
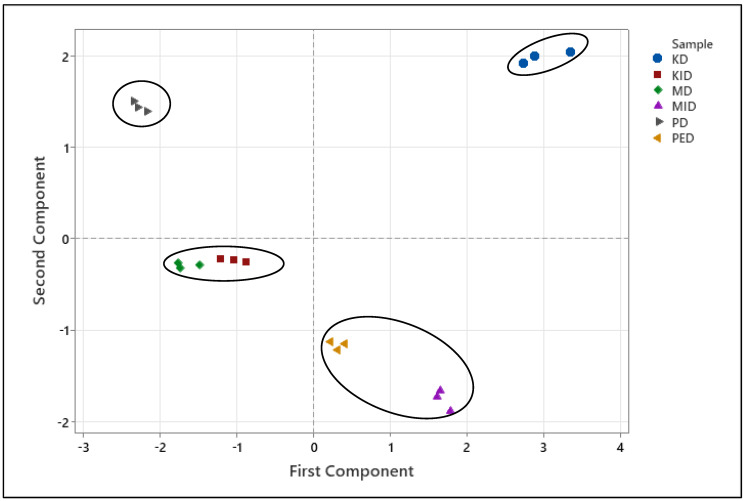
PCA score plot of dried fruits and vegetables (three replicates for each product). The ellipses only define the category position in the PCA score plot with no statistical meaning. MD = apple; PD = tomato; MID = blueberry; KID = kiwifruit; PED = peach; KD = kaki.

The health-promoting benefits associated with dried fruits and vegetables may be attributed to the synergistic and combined action of different phenolics (phytocomplex) rather than the individual effects of each molecule [27,61]. Therefore, the molecules were categorized into chemical classes to facilitate multivariate data analysis. The selected variables comprised four chemical classes, namely, benzoic acids, cinnamic acids, catechins, and flavonols, in addition to AOC (antioxidant capacity), HPLC TPs (total phenolics by HPLC), and TPC (total polyphenol content). Bartlett’s test of sphericity (*p* < 0.05) revealed significant statistical collinearity among all the considered variables, while the KMO index presented a level of 0.65. The two principal components explained 80.1% of the total variance (53.2% by PC1 and 26.9% by PC2). The score plot illustrated the positioning of the six samples (mean values obtained from three repetitions for each dried product) in the PC plane based on their nutraceutical properties and phenolic composition (Figure 3). The proximity of points on the plane indicated the similarity of the dried product phenolic profile and antioxidant properties [62,63]. Dried fruits and vegetables with similar phenolic composition were closely located, suggesting a strong correlation between the single phenolics, the total polyphenol content, and the overall antioxidant capacity. This information can be useful for understanding the traits of different dried products and their potential applications as health-promoting food items. The score plot showed the six dried products as divided into four groups [i.e., (i) tomato; (ii) kaki; (iii) apple + kiwifruit; (iv) peach + blueberry] based on their phenolic composition and antioxidant properties, confirming the statistical univariate results.

**Figure 4:**
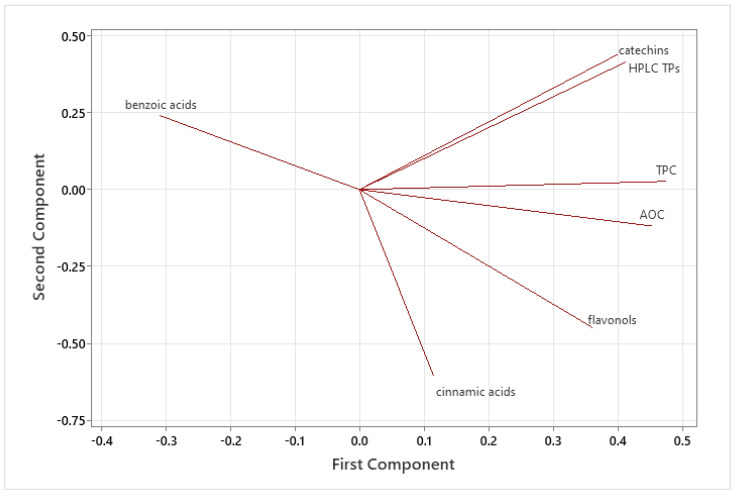
PCA loading plot of the considered variables.

The PCA loading plot (Figure 4) revealed interesting associations between several quantified phenolics and AOC in the analyzed dried products. An important correlation was highlighted among TPC, AOC, HPLC TPs, catechins, flavonols, and PC1. This issue suggested that these compounds may have a shared influence on the overall composition of the samples, confirming the results of Pearson’s correlation test. Additionally, there was a correlation between phenolic acids and PC2, indicating a potential link between these compounds. In any case, further analysis is necessary to provide more insight into these relationships and their implications.

In this study, the results confirmed that these phenolic molecules may play a crucial role in the potential health benefits of dried products, as already reported in the literature [7,12,15]. The considered classes, used as variables in the PCA, have been identified as having a good discriminating power among the different products, indicating their role in distinguishing among several dried vegetables and fruits. Specific phenolic groups (i.e., mainly catechins, followed by flavonols) have been identified as biomarkers, with significant differences in their contents among the different dried products. The proposed approach (chromatographic fingerprinting coupled to chemometrics) resulted in an effective tool for the quality control and valorization of these products. Indeed, the PCA showed that dried apples and kiwifruits were not characterized by specific phenolics, while dried tomato products were well defined by the quantification of benzoic acids, gallic acid, in particular, as well as dried peaches were distinguished from the other products by the evaluation of cinnamic acids (e.g., caffeic and p-coumaric acids). On the other hand, dried kaki products were mainly characterized by catechins, while dried blueberries showed high amounts of flavonols. This information could be used to differentiate and evaluate the quality of dried fruit products for many purposes, such as label certifications, and the product composition control may benefit from the application of these different markers. In any case, further analysis may be focused on the evaluation of dried fruits and vegetables with different origins and/or genotypes, also as commercial products, to fully demonstrate the applicability of this approach, as already shown for other food items derived from plant material [34,64].

## 3. Materials and Methods

### 3.1. Plant Material

Fruits were collected during the 2024 season from Azienda Agricola Albertengo, a commercial farm located in Revello (Cuneo Province, Italy). Horticultural crop remains (HCRs) were washed and then stored at 4 °C and 95% R.H. (relative humidity) until drying processes. Fruit edible parts were separated from the inedible ones.

The investigated dried fruits and vegetables were derived from six species: (i) apple, *Malus domestica* Borkh. (‘Golden Delicious’ cv); (ii) tomato, *Solanum lycopersicum* L. (‘San Marzano’ cv); (iii) blueberry, *Vaccinium corymbosum* L. (‘Duke’ cv); (iv) kiwifruit, *Actinidia chinensis* var. *deliciosa* (A. Chev.) C.F. Liang and A.R. Ferguson (‘Hayword’ cv); (v) peach, *Prunus persica* (L.) Batsch (‘Maycrest’ cv); and (vi) kaki, *Diospyros kaki* L.F. (‘Fuyu’ cv).

### 3.2. Freeze-Drying

Fresh, frozen plant material was freeze-dried, removing water by dehydration. This technique is based on the sublimation of ice in fruits by a hot air flow at medium temperature (35–40 °C) and low humidity (2–3%). The used freeze dryer (NWT-25, North West Technology, Cuneo, Italy) operated in the “vaporization chain system” utilizing the following settings: (i) a drying cycle time in a range from 6 to 48 h; (ii) a total capacity for each drying cycle of 40 Kg; and (iii) a range of 0.70–0.75 kW/h as the system power.

The small pieces and slices of fresh fruits and vegetables were frozen at different cooling rates in a freezer. Slow freezing (SF) was carried out in the freezer with minimal fan operation, by programming a gradual decrease in the freezing temperature according to the following sequence of parameters (air temperature and time): −4 °C/2 h, −7 °C/2 h, −10 °C/2 h, −12 °C/2 h, −15 °C/2 h, and −40 °C/2 h. Primary drying occurred when the chamber temperature reached −40 °C and the pressure was subsequently reduced to 10 Pa (a Pirani gauge was used as a vacuum gauge) to sublime the frozen water in the fruit directly from solid ice to water vapor. After primary drying was finished, secondary drying began by slowly increasing the shelf temperature to 35–40 °C while maintaining the pressure inside the freeze-dryer unchanged (63 Pa) throughout this process to remove any residual moisture. During freeze-drying, the conditions were selected to obtain a minimum moisture content of 5–10% for the different products. Changes in the mass of the dried samples were continuously recorded during drying with an accuracy of ±0.1 g. Once secondary drying was complete, the freeze-dried samples were removed and used for the phenolic extraction process.

### 3.3. Analytical Techniques and Protocols

HPLC solvents, chemical reagents, and standards are reported in the Supplementary Materials.

#### 3.3.1. Phenolic Extraction

For each species, 10 g of dried fruits were used (n = 3). The extraction of the main phenolic markers, selected for their health-promoting properties, was performed by adding 25 mL of a solution of 37% HCl:water:methanol at a 0.5:4.5:95 (*v*/*v*/*v*) ratio to the dried material. Maceration for 24 h was used as the extraction method. These extracts were filtered by a polytetrafluoroethylene (PTFE) membrane micro-filter (pore size: 0.45 μm). Then, they were stored at 4 °C and 95% R.H. until spectrophotometric and chromatographic analysis.

#### 3.3.2. Total Polyphenolic Content and Antioxidant Capacity

The Folin–Ciocalteu method [65] was carried out to evaluate the total polyphenolic content (TPC). TPC values were expressed as mg of gallic acid equivalents (GAE) per 100 g of dried weight (DW). Gallic acid (0.02–0.1 mg/mL as concentration range) was used for the standard calibration curve.

The Ferric Reducing Antioxidant Power (FRAP) assay [66] was performed for a chemical screening of antioxidant capacity (AOC) in the selected dried fruits. AOC results were expressed as millimoles of ferrous iron (Fe^2+^) equivalents per kilogram of DW. The calibration curve was performed with 100–1000 mmol/L solutions of FeSO_4_·7H_2_O.

A UV/Vis single-beam spectrophotometer (1600-PC, VWR International, Milan, Italy) was utilized to assess TPC at 760 nm and AOC at 595 nm, respectively.

#### 3.3.3. Quantification of Phenolic Compounds and Bioinformatic Prediction of Their Biological Targets

Phenolic markers were identified and characterized by high-performance liquid chromatography (HPLC) by an Agilent 1200 HPLC (Agilent Technologies, Santa Clara, CA, USA) coupled to a UV–Vis diode array detector (Agilent Technologies, Santa Clara, CA, USA).

The chromatographic conditions are fully detailed in the Supplementary Materials (Table S1). Polyphenols were separated by a Kinetex—C18 column (4.6 × 150 mm, 5 μm, Phenomenex, Torrance, CA, USA). Two HPLC methods were carried out following the analytical conditions already validated in previous studies [27,64], with a few modifications. The analyte quantification was assessed by an external standard calibration and expressed as mg per 100 g of DW. Specific wavelengths (330 nm and 280 nm) were considered for the identification and quantification of specific peaks. The scanning range was from 190 to 600 nm. The identification of all the molecules was achieved through a process of comparing and combining their retention times and UV–vis spectra with relative analytical standards, performed under the same chromatographic conditions.

For each considered dried product, the chemical structure of the main phenolics was subjected to a bioinformatic approach to predict the main potential biological targets in humans and confirm their health-promoting effects. The chemical structures were prepared by molecular design software (ACD/ChemSketch 12.0, Advanced Chemistry Development Inc., Toronto, ON, Canada) and then converted into SMILES (Simplified Molecular Input Line Entry System) format for the evaluation. The molecules in SMILES format were then analyzed by the Swiss Target Prediction web platform (http://www.swisstargetprediction.ch/, accessed on 20 September 2024), as reported in similar studies [26,48,67].

### 3.4. Statistical Analysis

For all the considered parameters (i.e., amounts of each phenolic compound, TPC, and AOC), the results, expressed as mean value ± standard deviation (SD), were compared with Tukey’s HSD post hoc comparison test at *p* < 0.05 (n = 3) after the application of a one-factor ANOVA test. Significant statistical differences for *p* < 0.05 were assessed by different letters in accordance with the results of the Tukey test. Moreover, Pearson’s coefficient (R) for *p* < 0.05 and *p* < 0.01 was utilized to investigate the positive and negative correlations, and their relative intensity, among each single phenolic compound, TPC, and AOC.

Multivariate analysis (MVA) was performed to observe the statistical differences among the considered dried products and their correlations depending on phenolic composition and antioxidant properties to confirm the analytical results obtained by univariate statistical tests and detect the most discriminant parameters to assess the data structure. Eighteen objects (six samples, three repetitions) and sixteen variables (single amount of the thirteen phenolic markers, total phenolics by HPLC (HPLC TPs), TPC, AOC) were included in the data matrix. Matrix data were column-centered by a scaling process (Z-score) before performing the MVA. Mean-centered data were then subjected to a Principal Component Analysis (PCA) after the application of Bartlett’s test of sphericity (BTS) and the Kaiser–Meyer–Olkin index (KMO). Original variables were recombined (correlation mode with a Varimax rotation) into principal components (PCs) corresponding to an eigenvalue greater than or equal to 1 with the explanation of at least 60% of the total original variance.

All statistical evaluations were performed with IBM SPSS Statistics 27.0 (IBM, Armonk, NY, USA), Minitab 20.4 (Minitab Inc., State College, PA, USA), and GraphPad Prism 10.0 software (GraphPad, Inc., La Jolla, CA, USA).

## 4. Conclusions

The purpose of this research was to determine the levels of phenolics and their potential health benefits in dried fruits and vegetables derived from the remains of crop production. The findings indicated that well-known freeze-dried items such as dried apples and peaches, as well as less common products such as dried tomatoes, blueberries, kiwifruits, and kaki, have preserved high concentrations of flavonoids, catechins, and phenolic acids, exhibiting strong antioxidant properties. For this reason, this study suggests that freeze-dried vegetables and fruits may be considered health-promoting foods, complementary to fresh products. This study also presents a bioinformatic approach to the human biological targets of the main phenolics quantified in freeze-dried products, even if more specific chemical studies and clinical trials are needed to fully understand and validate the health benefits of these products. This research contributes to the development of new freeze-dried products that may be widely available in the market, even if further research is needed to evaluate the safety for consumption and the quality of these products, and that may be a potential solution for (i) preventing post-harvest fruit losses in the agri-food industry by using crop remains, (ii) potentially increasing profits of farmers and growers who may directly utilize this technology for their purposes, and (iii) sustainably reducing agricultural waste.

## Figures and Tables

**Table 1:** The main traits of the dried fruits considered in this study.

Common Name	Species	Cultivar	ID Code	Appearance	Weight (g)
Apple	*Malus domestica* Borkh.	Golden Delicious	MD	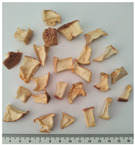	0.44 ± 0.14
Tomato	*Solanum lycopersicum* L.	San Marzano	PD	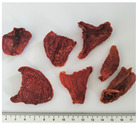	1.37 ± 0.36
Blueberry	*Vaccinium corymbosum* L.	Duke	MID	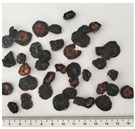	0.17 ± 0.05
Kiwifruit	*Actinidia chinensis* Planch.	Hayword	KID	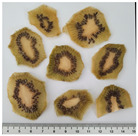	0.91 ± 0.33
Peach	*Prunus persica* (L.) Batsch	Maycrest	PED	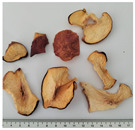	0.72 ± 0.19
Kaki	*Diospyros kaki* L.F.	Fuyu	KD	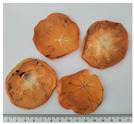	2.97 ± 0.40

**Table 2:** Total phenolic content (TPC) and antioxidant capacity (AOC) of the analyzed dried products.

	Antioxidant Capacity			Total Polyphenolic Content		
	(mmol Fe^2+^/kg DW)			(mgGAE/100 g DW)		
ID Code	Mean Value	SD	Tukey Test	Mean Value	SD	Tukey Test
MD	49.28	2.88	a	366.86	71.30	a
PD	58.41	2.24	b	415.60	52.43	ab
MID	80.43	0.02	c	1077.13	35.47	b
KID	64.52	4.94	b	428.25	4.21	ab
PED	74.25	2.37	c	521.10	10.19	ab
KD	79.05	0.21	c	1102.25	219.71	b

Results (n = 3) are detailed as mean value ± standard deviation (SD). Significant statistical differences for *p* < 0.05 are reported with different letters (a–c). GAE—gallic acid equivalent; DW—dried weight. MD = apple; PD = tomato; MID = blueberry; KID = kiwifruit; PED = peach; KD = kaki.

**Table 3:** Phenolic amounts of the analyzed dried products.

mg/100 g DW		ID Code	Mean	SD	Tukey Test
Cinnamic acids	caffeic acid	KD	n.d.	/	/
		KID	0.68	0.08	a
		MD	n.d.	/	/
		MID	0.53	0.02	a
		PD	0.46	0.01	a
		PED	15.98	0.25	b
	chlorogenic acid	KD	n.d.	/	/
		KID	1.69	0.24	b
		MD	n.d.	/	/
		MID	0.35	0.11	a
		PD	0.28	0.00	a
		PED	1.35	0.02	b
	p-coumaric acid	KD	0.59	0.03	a
		KID	n.d.	/	/
		MD	14.07	0.12	b
		MID	28.27	0.63	d
		PD	0.51	0.03	a
		PED	20.21	0.22	c
	ferulic acid	KD	2.81	0.05	ab
		KID	1.99	0.09	a
		MD	2.28	0.02	ab
		MID	3.70	0.63	b
		PD	2.92	0.20	ab
		PED	2.62	0.36	ab
Flavonols	hyperoside	KD	0.91	0.07	a
		KID	n.d.	/	/
		MD	n.d.	/	/
		MID	1.21	0.42	a
		PD	n.d.	/	/
		PED	n.d.	/	/
	isoquercitrin	KD	n.d.	/	/
		KID	n.d.	/	/
		MD	n.d.	/	/
		MID	n.d.	/	/
		PD	n.d.	/	/
		PED	n.d.	/	/
	quercetin	KD	18.22	0.15	b
		KID	16.58	0.33	a
		MD	16.91	0.19	a
		MID	19.98	0.36	b
		PD	n.d.	/	/
		PED	17.38	0.39	ab
	quercitrin	KD	n.d.	/	/
		KID	n.d.	/	/
		MD	n.d.	/	/
		MID	6.83	0.75	a
		PD	n.d.	/	/
		PED	n.d.	/	/
	rutin	KD	n.d.	/	/
		KID	n.d.	/	/
		MD	n.d.	/	/
		MID	0.26	0.16	a
		PD	8.68	0.27	b
		PED	n.d.	/	/
Benzoic acids	ellagic acid	KD	n.d.	/	/
		KID	0.24	0.02	a
		MD	4.45	0.09	d
		MID	3.89	0.23	c
		PD	5.60	0.19	e
		PED	1.17	0.11	b
	gallic acid	KD	n.d.	/	/
		KID	n.d.	/	/
		MD	n.d.	/	/
		MID	n.d.	/	/
		PD	12.11	1.87	a
		PED	n.d.	/	/
Catechins	(+)-catechin	KD	26.08	3.71	b
		KID	10.60	0.30	a
		MD	n.d.	/	/
		MID	n.d.	/	/
		PD	76.17	11.76	c
		PED	94.69	3.88	d
	(−)-epicatechin	KD	958.17	14.78	e
		KID	18.65	0.36	a
		MD	80.52	3.34	b
		MID	186.83	2.99	d
		PD	89.08	2.30	b
		PED	152.42	2.12	c
Total HPLC polyphenols	TOT	KD	1006.80	18.10	d
		KID	50.43	1.20	a
		MD	118.72	3.38	b
		MID	252.58	1.53	c
		PD	195.81	13.89	b
		PED	306.31	5.46	c

Results (n = 3) are detailed as mean value ± standard deviation (SD). Total HPLC polyphenols expressed the sum of the amounts of all the quantified markers for each dried product, according to the “multi-marker approach” [40]. Significant statistical differences for *p* < 0.05 are reported with different letters (a–e). DW—dried weight. MD = apple; PD = tomato; MID = blueberry; KID = kiwifruit; PED = peach; KD = kaki.

**Table 4:** Human molecular targets of the main phenolics quantified in the analyzed dried products.

Common Name	ID Code	Main Phenolics	Chemical Structure	Biological Target (*Homo sapiens*)
Apple	MD	p-Coumaric acid	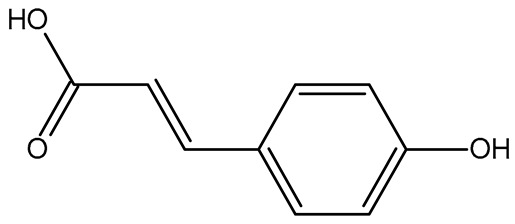	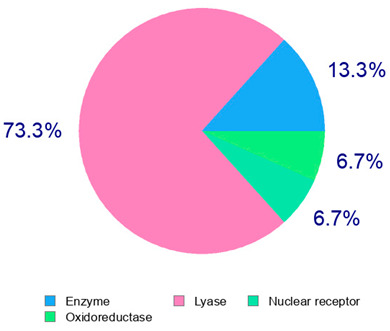
		Quercetin	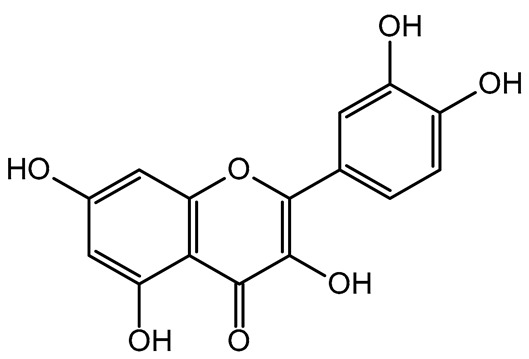	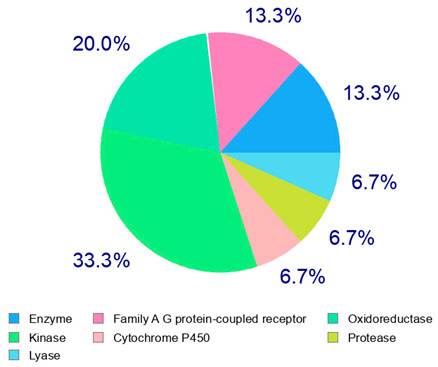
		Ellagic acid	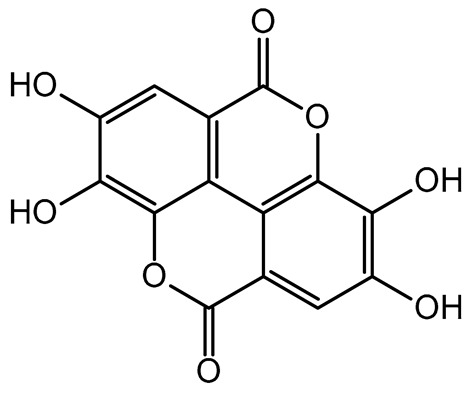	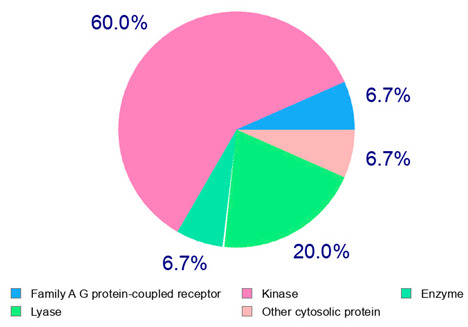
Tomato	PD	Ferulic acid	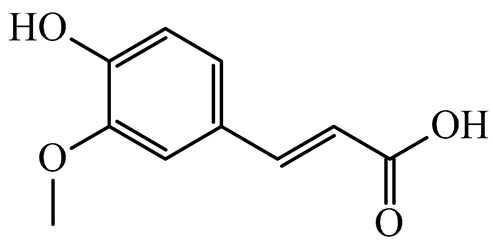	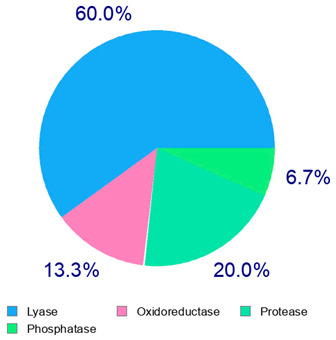
		Rutin	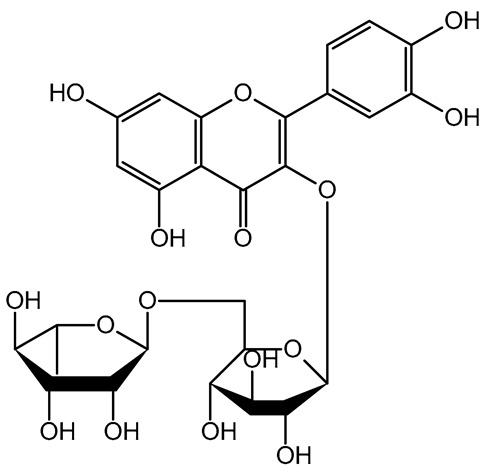	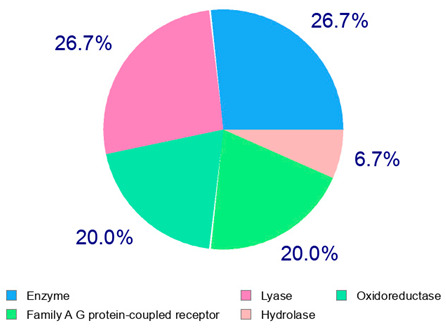
		Gallic acid	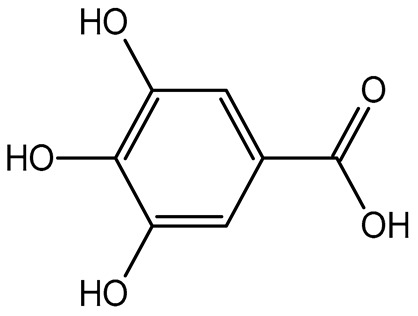	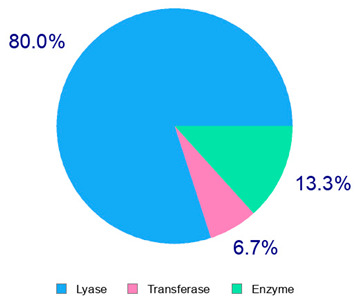
Blueberry	MID	p-Coumaric acid	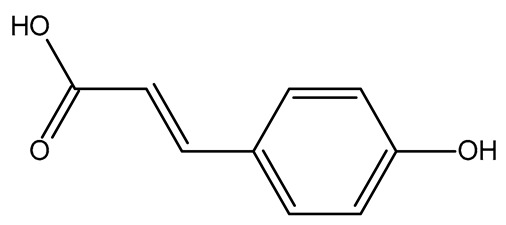	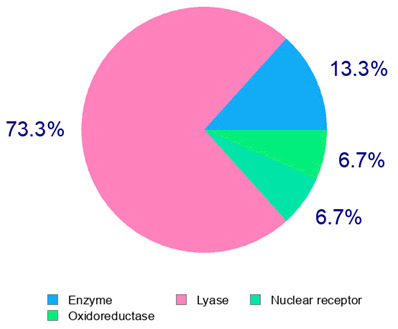
		Quercitrin	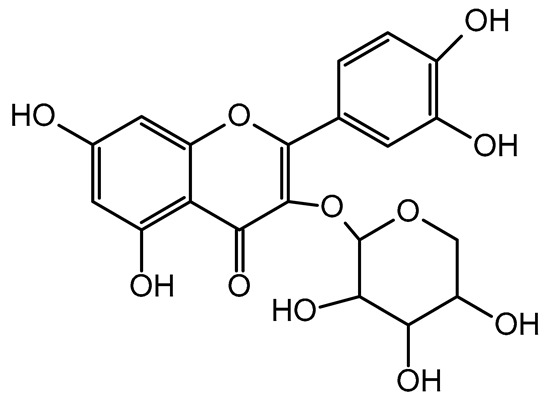	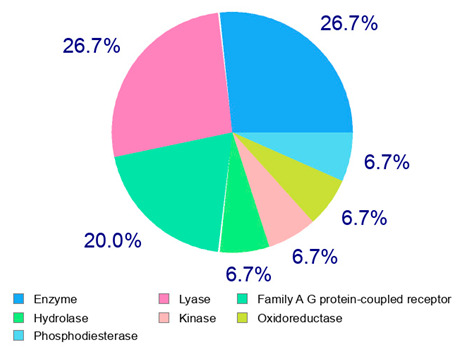
Kiwifruit	KID	Ferulic acid	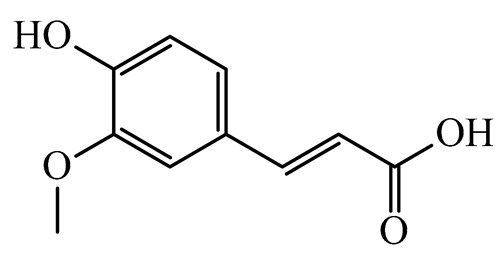	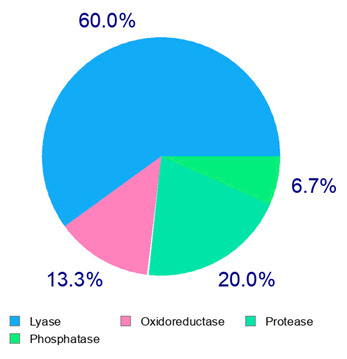
		Quercetin	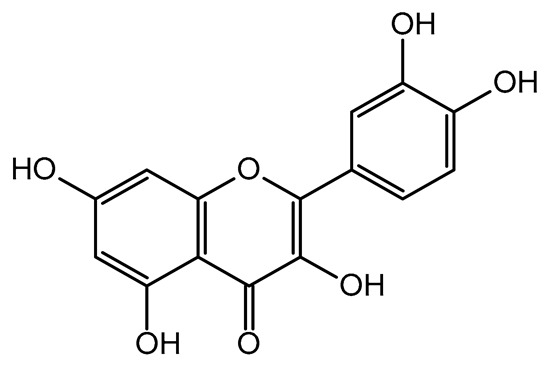	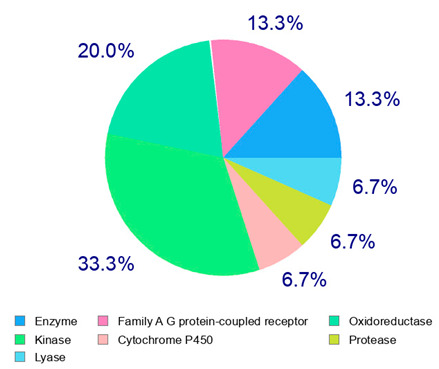
Peach	PED	Caffeic acid	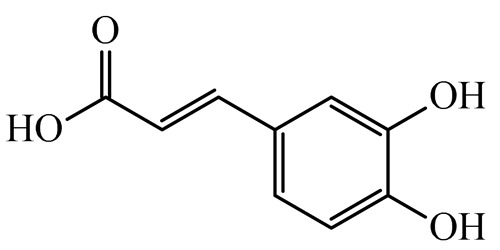	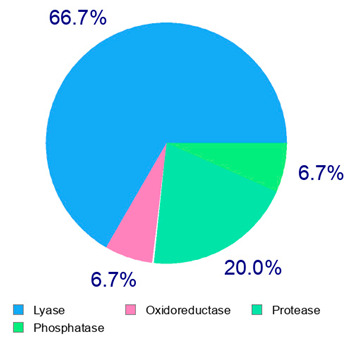
		p-Coumaric acid	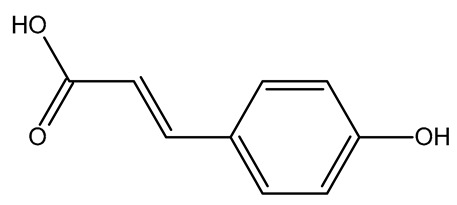	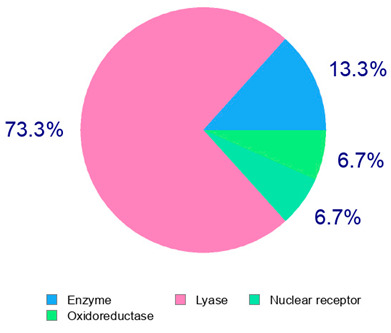
		Quercetin	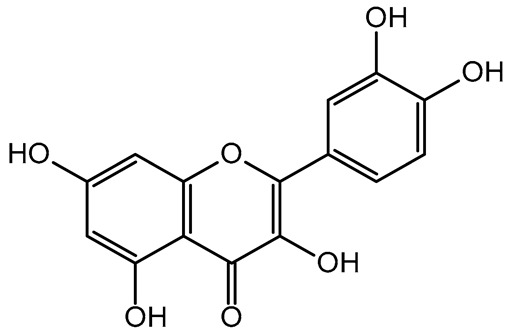	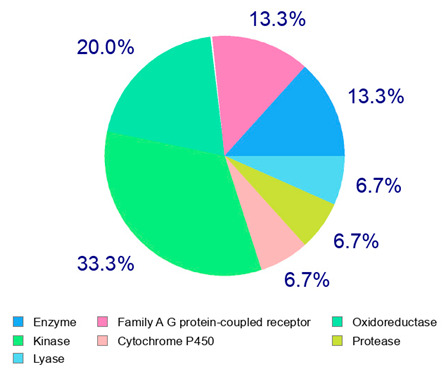
Kaki	KD	Ferulic acid	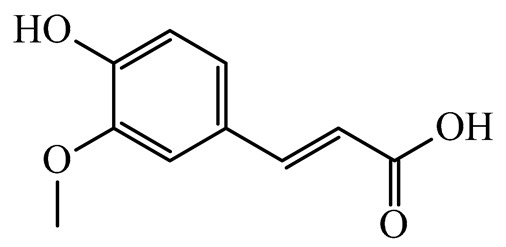	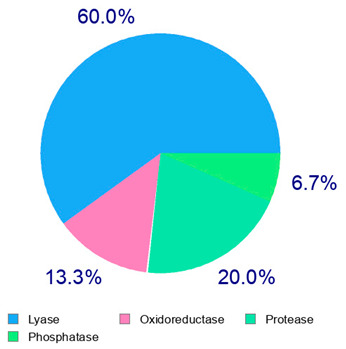
		Quercetin	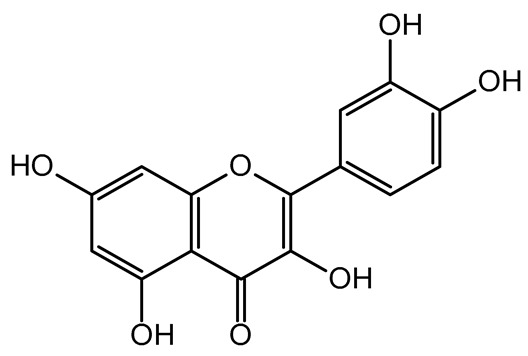	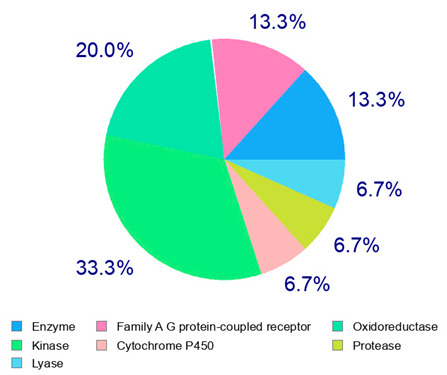

**Table 5:** Human molecular targets of the main catechins quantified in the analyzed dried products.

Phenolic Active Marker	Chemical Structure	Biological Target (*Homo sapiens*)	Sample (ID Code)
(+)-Catechin	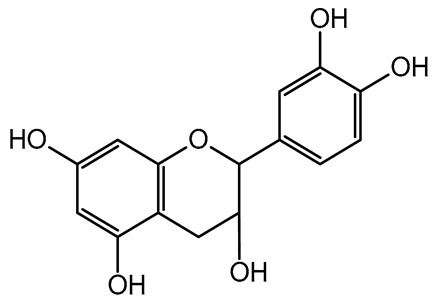	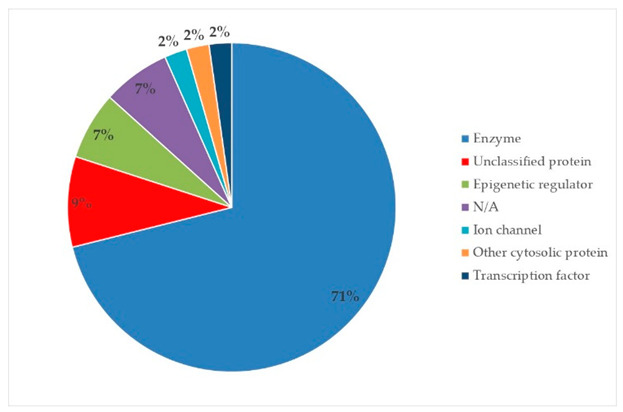	Tomato (PD) Kiwi (KID) Peach (PED) Kaki (KD)
(−)-Epicatechin	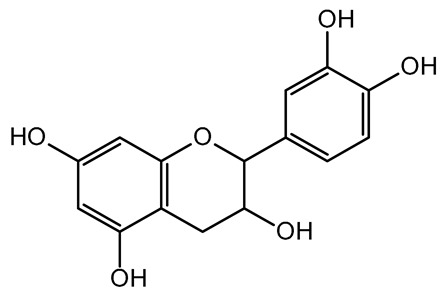	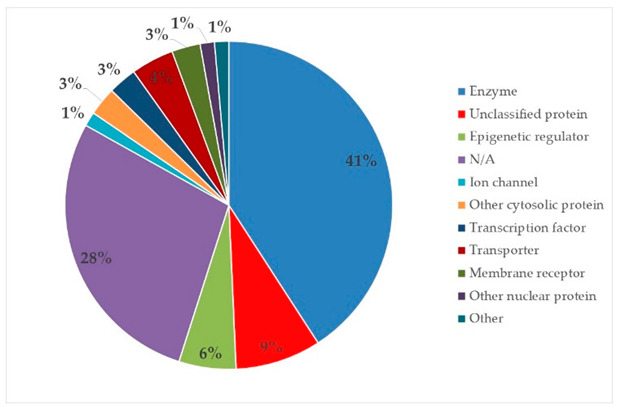	Apple (MD) Tomato (PD) Blueberry (MID) Kiwi (KID) Peach (PED) Kaki (KD)

## Data Availability

Data are not available in any public archive, nevertheless data can be shared with other interested researchers and authors by request.

## Supplementary Materials

The following supporting information can be downloaded at https://www.mdpi.com/article/10.3390/plants14020168/s1, Table S1. Chromatographic conditions of the used methods.

## References

[1] Salim N.S.M., Singh A., Raghavan V. (2017). Potential utilization of fruit and vegetable wastes for food through drying or extraction techniques. Nov. Tech. Nutr. Food Sci..

[2] Tan C.H., Hii C.L., Borompichaichartkul C., Phumsombat P., Kong I., Pui L.P. (2022). Valorization of fruits, vegetables, and their by-products: Drying and bio-drying. Dry. Technol..

[3] Galanakis C.M. (2013). Emerging technologies for the production of nutraceuticals from agricultural by-products: A viewpoint of opportunities and challenges. Food Bioprod. Process..

[4] Đilas S., Čanadanović-Brunet J., Ćetković G. (2009). By-products of fruits processing as a source of phytochemicals. Chem. Ind. Chem. Eng. Q..

[5] Sagar V., Suresh Kumar P. (2010). Recent advances in drying and dehydration of fruits and vegetables: A review. J. Food Sci. Technol..

[6] Hasan M.U., Malik A.U., Ali S., Imtiaz A., Munir A., Amjad W., Anwar R. (2019). Modern drying techniques in fruits and vegetables to overcome postharvest losses: A review. J. Food Process. Preserv..

[7] Alasalvar C., Chang S.K., Kris-Etherton P.M., Sullivan V.K., Petersen K.S., Guasch-Ferré M., Jenkins D.J. (2023). Dried Fruits: Bioactives, Effects on Gut Microbiota, and Possible Health Benefits—An Update. Nutrients.

[8] Alasalvar C., Salvadó J.-S., Ros E. (2020). Bioactives and health benefits of nuts and dried fruits. Food Chem..

[9] Donno D., Mellano M.G., Raimondo E., Cerutti A.K., Prgomet Ž., Beccaro G.L. (2016). Influence of applied drying methods on phytochemical composition in fresh and dried goji fruits by HPLC fingerprint. Eur. Food Res. Technol..

[10] Delgado T., Pereira J.A., Ramalhosa E., Casal S. (2017). Comparison of different drying methods on the chemical and sensory properties of chestnut (*Castanea sativa* M.) slices. Eur. Food Res. Technol..

[11] Zhang L., Wang Z., Shi G., Yang H., Wang X., Zhao H., Zhao S. (2018). Effects of drying methods on the nutritional aspects, flavor, and processing properties of Chinese chestnuts. J. Food Sci. Technol..

[12] Donno D., Mellano M.G., Riondato I., De Biaggi M., Andriamaniraka H., Gamba G., Beccaro G.L. (2019). Traditional and Unconventional Dried Fruit Snacks as a Source of Health-Promoting Compounds. Antioxidants.

[13] Rybicka I., Kiewlicz J., Kowalczewski P.Ł., Gliszczyńska-Świgło A. (2021). Selected dried fruits as a source of nutrients. Eur. Food Res. Technol..

[14] Alasalvar C., Shahidi F. (2013). Composition, phytochemicals, and beneficial health effects of dried fruits: An overview. Dried Fruits: Phytochemicals and Health Effects.

[15] Vinson J.A., Zubik L., Bose P., Samman N., Proch J. (2005). Dried fruits: Excellent in vitro and in vivo antioxidants. J. Am. Coll. Nutr..

[16] Chang S.K., Alasalvar C., Shahidi F. (2016). Review of dried fruits: Phytochemicals, antioxidant efficacies, and health benefits. J. Funct. Foods.

[17] Sadler M.J., Gibson S., Whelan K., Ha M.-A., Lovegrove J., Higgs J. (2019). Dried fruit and public health–what does the evidence tell us?. Int. J. Food Sci. Nutr..

[18] Nemzer B., Vargas L., Xia X., Sintara M., Feng H. (2018). Phytochemical and physical properties of blueberries, tart cherries, strawberries, and cranberries as affected by different drying methods. Food Chem..

[19] Ashebir D., Jezik K., Weingartemann H., Gretzmacher R. (2009). Change in color and other fruit quality characteristics of tomato cultivars after hot-air drying at low final-moisture content. Int. J. Food Sci. Nutr..

[20] Kim Y., Hertzler S.R., Byrne H.K., Mattern C.O. (2008). Raisins are a low to moderate glycemic index food with a correspondingly low insulin index. Nutr. Res..

[21] Widanagamage R.D., Ekanayake S., Welihinda J. (2009). Carbohydrate-rich foods: Glycaemic indices and the effect of constituent macronutrients. Int. J. Food Sci. Nutr..

[22] Spiegel M., Kapusta K., Kołodziejczyk W., Saloni J., Żbikowska B., Hill G.A., Sroka Z. (2020). Antioxidant activity of selected phenolic acids–ferric reducing antioxidant power assay and QSAR analysis of the structural features. Molecules.

[23] Hu W., Sarengaowa, Guan Y., Feng K. (2022). Biosynthesis of phenolic compounds and antioxidant activity in fresh-cut fruits and vegetables. Front. Microbiol..

[24] Osman M.A., Mahmoud G.I., Shoman S.S. (2020). Correlation between total phenols content, antioxidant power and cytotoxicity. Biointerface Res. Appl. Chem..

[25] Zeb A. (2020). Concept, mechanism, and applications of phenolic antioxidants in foods. J. Food Biochem..

[26] Fawzi Mahomoodally M., Picot-Allain M.C.N., Zengin G., Llorent-Martínez E.J., Abdullah H.H., Ak G., Senkardes I., Chiavaroli A., Menghini L., Recinella L. (2020). Phytochemical analysis, network pharmacology and in silico investigations on Anacamptis pyramidalis tuber extracts. Molecules.

[27] Donno D., Turrini F., Farinini E., Mellano M.G., Boggia R., Beccaro G.L., Gamba G. (2024). Chestnut Episperm as a Promising Natural Source of Phenolics from Agri-Food Processing by-Products: Optimisation of a Sustainable Extraction Protocol by Ultrasounds. Agriculture.

[28] Sadowska-Bartosz I., Bartosz G. (2022). Evaluation of the antioxidant capacity of food products: Methods, applications and limitations. Processes.

[29] Tiranakwit T., Puangpun W., Tamprasit K., Wichai N., Siriamornpun S., Srisongkram T., Weerapreeyakul N. (2023). Phytochemical Screening on Phenolic, Flavonoid Contents, and Antioxidant Activities of Six Indigenous Plants Used in Traditional Thai Medicine. Int. J. Mol. Sci..

[30] Schaich K.M., Tian X., Xie J. (2015). Reprint of “Hurdles and pitfalls in measuring antioxidant efficacy: A critical evaluation of ABTS, DPPH, and ORAC assays”. J. Funct. Foods.

[31] Alvarez-Parrilla E., de la Rosa L.A., González-Aguilar G.A., Ayala-Zavala J.F. (2013). Phytochemical Composition and Health Aspects of Peach Products. Dried Fruits: Phytochemicals and Health Effects.

[32] Song X.-D., Mujumdar A.S., Law C.-L., Fang X.-M., Peng W.-J., Deng L.-Z., Wang J., Xiao H.-W. (2020). Effect of drying air temperature on drying kinetics, color, carotenoid content, antioxidant capacity and oxidation of fat for lotus pollen. Dry. Technol..

[33] Sousa A.D., Ribeiro P.R.V., Canuto K.M., Zocolo G.J., Pereira R.d.C.A., Fernandes F.A.N., Sousa de Brito E. (2018). Drying kinetics and effect of air-drying temperature on chemical composition of Phyllanthus amarus and Phyllanthus niruri. Dry. Technol..

[34] Donno D., Fabro M., Mellano M.G., Gamba G., Fioccardi A., Beccaro G.L. (2022). Integrating Traditional Wheat-Based Foods with High Health Value Flours: *Castanea* spp. Agro-Biodiversity in Bakery Products. Agriculture.

[35] Fioccardi A., Donno D., Razafindrakoto Z.R., Gamba G., Beccaro G.L. (2022). First phytochemical study of six tree and shrub species with high health-promoting potential from Madagascar: Innovative uses for food and medicinal applications. Sci. Hortic..

[36] Rupasinghe H.V., Joshi A.P. (2013). Phytochemicals and health benefits of dried apple snacks. Dried Fruits: Phytochemicals and Health Effects.

[37] Asami D.K., Hong Y.-J., Barrett D.M., Mitchell A.E. (2003). Comparison of the total phenolic and ascorbic acid content of freeze-dried and air-dried marionberry, strawberry, and corn grown using conventional, organic, and sustainable agricultural practices. J. Agric. Food Chem..

[38] Meng J., Fang Y., Zhang A., Chen S., Xu T., Ren Z., Han G., Liu J., Li H., Zhang Z. (2011). Phenolic content and antioxidant capacity of Chinese raisins produced in Xinjiang Province. Food Res. Int..

[39] Değirmencioğlu N., Gürbüz O., Karatepe G.E., Irkin R. (2017). Influence of hot air drying on phenolic compounds and antioxidant capacity of blueberry (Vaccinium myrtillus) fruit and leaf. J. Appl. Bot. Food Qual..

[40] Mok D.K.W., Chau F.T. (2006). Chemical information of Chinese medicines: A challenge to chemist. Chemom. Intell. Lab. Syst..

[41] Li F., Li S., Li H.-B., Deng G.-F., Ling W.-H., Wu S., Xu X.-R., Chen F. (2013). Antiproliferative activity of peels, pulps and seeds of 61 fruits. J. Funct. Foods.

[42] Alasalvar C., Shahidi F. (2013). Dried Fruits: Phytochemicals and Health Effects.

[43] Kaya A., Aydın O., Kolaylı S. (2010). Effect of different drying conditions on the vitamin C (ascorbic acid) content of Hayward kiwifruits (*Actinidia deliciosa* Planch). Food Bioprod. Process..

[44] Chen F., Zhang M., Mujumdar A.S., Guo C., Yu D. (2021). Comparative analysis of composition and hygroscopic properties of infrared freeze-dried blueberries, cranberries and raspberries. Dry. Technol..

[45] Wang Y., Gallegos J.L., Haskell-Ramsay C., Lodge J.K. (2022). Effects of blueberry consumption on cardiovascular health in healthy adults: A cross-over randomised controlled trial. Nutrients.

[46] Mei S., Chen X. (2023). Combination of HPLC-orbitrap-MS/MS and network pharmacology to identify the anti-inflammatory phytochemicals in the coffee leaf extracts. Food Front..

[47] Gamba G., Donno D., Mellano M.G., Riondato I., De Biaggi M., Randriamampionona D., Beccaro G.L. (2020). Phytochemical Characterization and Bioactivity Evaluation of Autumn Olive (*Elaeagnus umbellata* Thunb.) Pseudodrupes as Potential Sources of Health-Promoting Compounds. Appl. Sci..

[48] Fioccardi A., Donno D., Razafindrakoto Z.R., Tombozara N., Henintsoa S., Mahitasoa E., Torti V., Solofoniaina M., Rosso L., Gamba G. (2024). Assessing a “Least-Concern” Red List Tree Species from Madagascar Used in Traditional Medicine: Morella spathulata (Myricaceae) Phyto-Compounds and Anti-Inflammatory Properties. Plants.

[49] Galmés S., Reynés B., Palou M., Palou-March A., Palou A. (2021). Absorption, distribution, metabolism, and excretion of the main olive tree phenols and polyphenols: A literature review. J. Agric. Food Chem..

[50] Schneider I., Bucar F. (2005). Lipoxygenase inhibitors from natural plant sources. Part 1: Medicinal plants with inhibitory activity on arachidonate 5-lipoxygenase and 5-lipoxygenase [sol] cyclooxygenase. Phytother. Res. Int. J. Devoted Pharmacol. Toxicol. Eval. Nat. Prod. Deriv..

[51] Occhipinti R., Boron W.F. (2019). Role of carbonic anhydrases and inhibitors in acid–base physiology: Insights from mathematical modeling. Int. J. Mol. Sci..

[52] Lee S.-H., Griffiths J.R. (2020). How and why are cancers acidic? Carbonic anhydrase IX and the homeostatic control of tumour extracellular pH. Cancers.

[53] Seko D., Fujita R., Kitajima Y., Nakamura K., Imai Y., Ono Y. (2020). Estrogen receptor β controls muscle growth and regeneration in young female mice. Stem Cell Rep..

[54] Lee D., Hong J.H. (2020). The fundamental role of bicarbonate transporters and associated carbonic anhydrase enzymes in maintaining ion and pH homeostasis in non-secretory organs. Int. J. Mol. Sci..

[55] Mboge M.Y., Combs J., Singh S., Andring J., Wolff A., Tu C., Zhang Z., McKenna R., Frost S.C. (2021). Inhibition of carbonic anhydrase using SLC-149: Support for a noncatalytic function of CAIX in breast cancer. J. Med. Chem..

[56] Aleti G., Troyer E.A., Hong S. (2023). G protein-coupled receptors: A target for microbial metabolites and a mechanistic link to microbiome-immune-brain interactions. Brain Behav. Immun.-Health.

[57] Mohos V., Fliszár-Nyúl E., Ungvári O., Kuffa K., Needs P.W., Kroon P.A., Telbisz Á., Özvegy-Laczka C., Poór M. (2020). Inhibitory effects of quercetin and its main methyl, sulfate, and glucuronic acid conjugates on cytochrome P450 enzymes, and on OATP, BCRP and MRP2 transporters. Nutrients.

[58] Huang M., Wu F., Zuo X., Liu J., Yu W., Xie R., Liu G., Tan Q., Wang Q., Liang Y. (2024). Quercetin Simultaneously Inhibited Cytochrome P450 and P-Glycoprotein to Improve the Pharmacokinetics of Osthole in Rat Plasma. Rev. Bras. Farmacogn..

[59] Messire G., Serreau R., Berteina-Raboin S. (2023). Antioxidant effects of catechins (EGCG), andrographolide, and curcuminoids compounds for skin protection, cosmetics, and dermatological uses: An update. Antioxidants.

[60] Bae J., Kim N., Shin Y., Kim S.-Y., Kim Y.-J. (2020). Activity of catechins and their applications. Biomed. Dermatol..

[61] Donno D., Turrini F., Boggia R., Guido M., Gamba G., Mellano M.G., Riondato I., Beccaro G.L. (2020). Sustainable extraction and use of natural bioactive compounds from the waste management process of *Castanea* spp. bud-derivatives: The finnover project. Sustainability.

[62] Beccaro G.L., Donno D., Lione G.G., De Biaggi M., Gamba G., Rapalino S., Riondato I., Gonthier P., Mellano M.G. (2020). *Castanea* spp. Agrobiodiversity Conservation: Genotype Influence on Chemical and Sensorial Traits of Cultivars Grown on the Same Clonal Rootstock. Foods.

[63] Esslinger S., Riedl J., Fauhl-Hassek C. (2014). Potential and limitations of non-targeted fingerprinting for authentication of food in official control. Food Res. Int..

[64] Donno D., Mellano M.G., Carini V., Bergamasco E., Gamba G., Beccaro G.L.J.A. (2023). Application of Traditional Cooking Methods in Chestnut Processing: Effects of Roasting and Boiling on Secondary Metabolites and Antioxidant Capacity in *Castanea* spp. Fruits. Agriculture.

[65] Singleton V.L., Orthofer R., Lamuela-Raventos R.M. (1999). Analysis of total phenols and other oxidation substrates and antioxidants by means of folin-ciocalteu reagent. Methods Enzymol..

[66] Benzie I.F., Strain J.J. (1999). Ferric reducing/antioxidant power assay: Direct measure of total antioxidant activity of biological fluids and modified version for simultaneous measurement of total antioxidant power and ascorbic acid concentration. Methods Enzymol..

[67] Gu L., Lu J., Li Q., Wu N., Zhang L., Li H., Xing W., Zhang X. (2020). A network-based analysis of key pharmacological pathways of Andrographis paniculata acting on Alzheimer’s disease and experimental validation. J. Ethnopharmacol..

