# Supplementary material for: Freeze-Drying for the Reduction of Fruit and Vegetable Chain Losses: A Sustainable Solution to Produce Potential Health-Promoting Food Applications
Source: Plants (Basel). 2025 Jan 9;14(2):168. doi: 10.3390/plants14020168 (PMC11768221; doi:10.3390/plants14020168)
Supplement: Supplementary file 1 [file plants-14-00168-s001.zip › plants-3332916-supplementary.pdf]

plants

MDPI

Article

Freeze-Drying for the Reduction of Fruit and Vegetable Chain Losses: A Sustainable Solution to Produce Potential Health-Promoting Food Applications

Dario Donno [1,*], Giovanna Neirotti [1], Annachiara Fioccardi [1], Zoarilala Rinah Razafindrakoto [2], Nantenaina Tombozara [2], Maria Gabriella Mellano [1], Gabriele Loris Beccaro [1] and Giovanni Gamba [1]

[1] Department of Agriculture, Forestry and Food Science, University of Torino, Largo Braccini 2, 10095 Grugliasco, Italy; giovanna.neirotti@unito.it (G.N.); annachiara.fioccardi@unito.it (A.F.); gabriella.mellano@unito.it (M.G.M.); gabriele.beccaro@unito.it (G.L.B.); giovanni.gamba@unito.it (G.G.)

[2] Institut Malgache de Recherches Appliquées, B.P. 3833, Antananarivo 101, Madagascar; zo_ari_lala@yahoo.fr (Z.R.R.); nzara89@gmail.com (N.T.)

* Correspondence: dario.donno@unito.it

Supplementary Materials

Solvents, Chemicals, and Standards

Analytical HPLC grade solvents (acetonitrile, methanol, and formic acid), reagents for HPLC buffer (potassium dihydrogen phosphate and phosphoric acid) were purchased from Fluka Biochemika (Buchs, Switzerland) and Sigma–Aldrich (St Louis, MO, USA).

All polyphenolic standards (caffeic acid, chlorogenic acid, coumaric acid, ferulic acid, hyperoside, isoquercitrin, quercetin, quercitrin, rutin, ellagic acid, gallic acid, catechin, epicatechin, castalagin, vescalagin) were purchased from Sigma–Aldrich. Milli-Q ultrapure water was produced by Sartorius Stedim Biotech mod. Arium (Sartorius, Göettingen, Germany).

Stock solutions of cinnamic acids and flavonols with a concentration of 1.0 mg mL^{-1} were prepared in methanol. From these solutions, four calibration standards (1000 ppm, 50 ppm, 250 ppm, 125 ppm) were prepared by dilution with methanol; stock solutions of benzoic acids, tannins, and catechins with a concentration of 1.0 mg mL^{-1} were prepared in a solution of 95% methanol and 5% water. From these solutions, four calibration standards were prepared by dilution with 50% methanol-water.

 https://doi.org/10.3390/plants14020168

Table S1. Chromatographic conditions of the used methods.

Method	Compounds of Interest	Stationary Phase	Mobile Phase	Flow (mL min^{-1})	Wavelength (nm)
A	cinnamic acids, flavonols	KINETEX—C18 column (4.6 × 150 mm, 5 µm)	A: 10 mM KH_2PO_4/H_3PO_4, pH = 2.8<br>B: CH_3CN	1.5	330
B	benzoic acids, catechins, Tannins	KINETEX—C18 column (4.6 × 150 mm, 5 µm)	A: H_2O/CH_3OH/HCOOH (5:95:0.1 v/v/v), pH = 2.5<br>B: CH_3OH/HCOOH (100:0.1 v/v)	0.6	280

Method A—gradient analysis: 5% B to 21% B in 17 min + 21% B in 3 min (2 min conditioning time). Method B—gradient analysis: 3% B to 85% B in 22 min + 85% B in 1 min (2 min conditioning time).
